# Safety, biodistribution and viral shedding of oncolytic vaccinia virus TG6002 administered intravenously in healthy beagle dogs

**DOI:** 10.1038/s41598-021-81831-2

**Published:** 2021-01-26

**Authors:** Jérémy Béguin, Murielle Gantzer, Isabelle Farine, Johann Foloppe, Bernard Klonjkowski, Christelle Maurey, Éric Quéméneur, Philippe Erbs

**Affiliations:** 1 Transgene, Illkirch-Graffenstaden, France; 2 UMR Virologie, INRA, Ecole Nationale Vétérinaire d’Alfort, ANSES, Université Paris-Est, Maisons-Alfort, France; 3 Department of Internal Medicine, Ecole Nationale Vétérinaire d’Alfort, Université Paris-Est, Maisons-Alfort, France

**Keywords:** Drug development, Targeted therapies, Drug safety

## Abstract

Oncolytic virotherapy is an emerging strategy that uses replication-competent viruses to kill tumor cells. We have reported the oncolytic effects of TG6002, a recombinant oncolytic vaccinia virus, in preclinical human xenograft models and canine tumor explants. To assess the safety, biodistribution and shedding of TG6002 administered by the intravenous route, we conducted a study in immune-competent healthy dogs. Three dogs each received a single intravenous injection of TG6002 at 10^5^ PFU/kg, 10^6^ PFU/kg or 10^7^ PFU/kg, and one dog received three intravenous injections at 10^7^ PFU/kg. The injections were well tolerated without any clinical, hematological or biochemical adverse events. Viral genomes were only detected in blood at the earliest sampling time point of one-hour post-injection at 10^7^ PFU/kg. Post mortem analyses at day 35 allowed detection of viral DNA in the spleen of the dog which received three injections at 10^7^ PFU/kg. Viral genomes were not detected in the urine, saliva or feces of any dogs. Seven days after the injections, a dose-dependent antibody mediated immune response was identified. In conclusion, intravenous administration of TG6002 shows a good safety profile, supporting the initiation of clinical trials in canine cancer patients as well as further development as a human cancer therapy.

## Introduction

Oncolytic viruses (OV) are an emerging class of antitumor therapies^1–4^. OV are designed to replicate selectively within, and subsequently lyse, cancer cells. In addition to direct oncolysis, OV can induce systemic antitumor immune responses^5^. To improve tumor lysis, OV can be designed to express immunostimulatory transgenes, antiangiogenic proteins or suicide genes^6^. Although several OV have been tested in clinical trials, only one genetically engineered herpes simplex virus (talimogene laherparepvec, Imlygic, Amgen Europe B.V., Breda, Netherlands) has been approved by the European Medicines Agency and the US Food and Drug Administration, for the treatment of unresectable melanoma^7,8^. Vaccinia virus (VACV) is one of many OV under investigation. Pexa-Vec (pexastimogene devacirepvec, JX-594, SillaJen Biotherapeutics, Seoul, South Korea) a thymidine kinase deleted Wyeth strain of VACV expressing the GM-CSF cytokine to activate immune cells at the tumor site has shown promising results in some phase I and phase II trials^9–14^.

Several spontaneous canine cancers, such as mammary cancers or invasive urothelial carcinoma, appear to be relevant models of human cancers. Indeed, clinical presentation, hormonal etiology, environment, histological features, molecular profiles (steroid hormone receptors, proliferation markers, epidermal growth factor, p53 suppressor gene mutations, cyclooxygenases or metalloproteinases), response and resistance to therapy are quite similar^15–22^. Moreover, the increased prevalence noticed in veterinary medicine facilitates timely completion of clinical studies^23–25^. Thereby, the evaluation of the potency of oncolytic viruses on spontaneous canine cancers should be valuable for assessing their potential benefit in human medicine.

TG6002 is a Copenhagen strain of VACV with targeted deletions of two genes, *thymidine kinase* (*TK; J2R*) and a subunit of the *ribonucleotide reductase* (*RR; I4L*), to enhance tumor specificity^26^. TG6002 is armed with the suicide gene *FCU1* which encodes a bifunctional chimeric protein that catalyzes the conversion of 5-fluorocytosine (5-FC) into the toxic metabolites 5-fluorouracil (5-FU) and 5-fluorouridine monophosphate (5-FUMP)^27^. Expression of the *FCU1* gene by the virus allows targeted chemotherapy within the tumor^26^. In murine xenograft models of hepatocarcinoma and colorectal cancer treated intravenously with TG6002 and with oral 5-FC, a significant reduction of tumor size and an intratumoral production of 5-FU were reported^26^. Moreover, systemic treatment with a *TK-RR*-deficient Western Reserve VACV expressing the *FCU1* gene in a mouse orthotopic model of renal carcinoma was associated with infiltration of CD8^+^ T lymphocytes and a decrease in the proportion of infiltrating Treg lymphocytes into the tumor, thus modifying the ratio of CD8^+^/CD4^+^ Treg lymphocytes in favor of CD8^+^ cytotoxic T cells^28^. Spontaneous canine tumors have been shown to be relevant models for human oncology^20,29–32^. TG6002 has been shown to replicate and to exert oncolytic potency in canine cell lines and canine xenograft model^33^. The lytic properties of TG6002 were tested on canine mammary tumor explants. In vitro infection of canine mammary carcinoma biopsies with TG6002 led to tumor necrosis and the conversion of 5-FC into 5-FU^33^.

A study on healthy dogs receiving intramuscular injections of TG6002 demonstrated a safety profile and the absence of viral shedding^34^. The intratumoral route has been favored for a long time in oncolytic virotherapy. However, this route has one major shortcoming in that it focuses on treatment of non-metastatic accessible tumors. Even if an abscopal effect has been reported in murine models, only limited data of distant effects are available in human medicine^35–40^. To overcome the drawback of the intratumoral route, the intravenous route has been considered^41,42^. The intravenous route is expected to target inaccessible tumors and treat both the primary tumor and any other diagnosed or undiagnosed metastatic disease. However, the intravenous route may lead to a stronger immune response against the OV. Previous study on mice treated with intravenous injections of TG6002, revealed the development of pock lesions on the tail nine days after treatment^26^. No other adverse events were observed. Although the host immune system’s activation plays a role in OV mediated tumor destruction, innate and adaptive immune responses can instigate clearance of OV and thereby limit oncolytic activity^43^. Oncolytic virus delivery by the intravenous route to tumor sites can be impeded by specific OV antibodies, neutralizing antibodies, complement proteins, splenic or hepatic sequestration, transfer into and throughout the tumor, cellular antiviral responses and destruction of infected tumor cells by cells of the innate immune system^43–46^. Thus, the characterization of both biodistribution and immune response is necessary to assess OV efficacy after intravenous administration. Intratumoral delivery can be increased by adjunctive technics. Indeed, ultrasound mediated cavitation has shown efficacy to improve the intratumoral delivery of TG6002 after systemic administration^47^.

Biosafety is a major concern with OV for both patients and the environment. Indeed, VACV infection is characterized by the development of cutaneous pock lesions that participate in the viral shedding^48^. Mucocutaneous pustules have been reported after intratumoral or intravenous attenuated oncolytic VACV injections in patients with cancer^9,11,14,49–52^. Environmental viral shedding is also a major issue as VACV can remain infectious for a long period in excreta^53–55^.

The first objective of the study was to assess the safety profile and viral shedding following intravenous injections of escalating doses of TG6002 in healthy dogs. The second objective was to evaluate immune responses induced by TG6002 injections in healthy dogs.

## Results

### Clinical toxicity and adverse events

**Figure 1:**
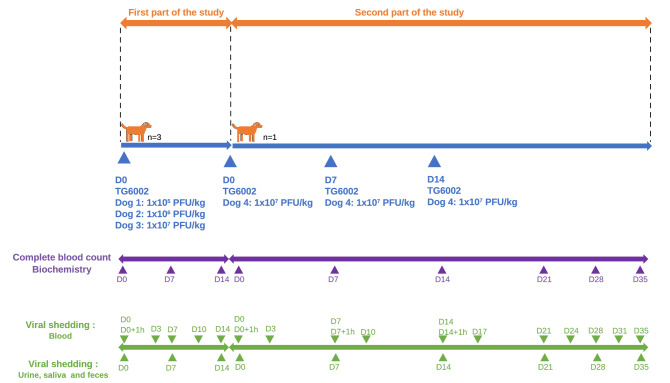
Study chart and sample collection for safety evaluation of intravenous administration of TG6002 in healthy dogs.

The schedule of the study is represented in Fig. 1.

**Figure 2:**
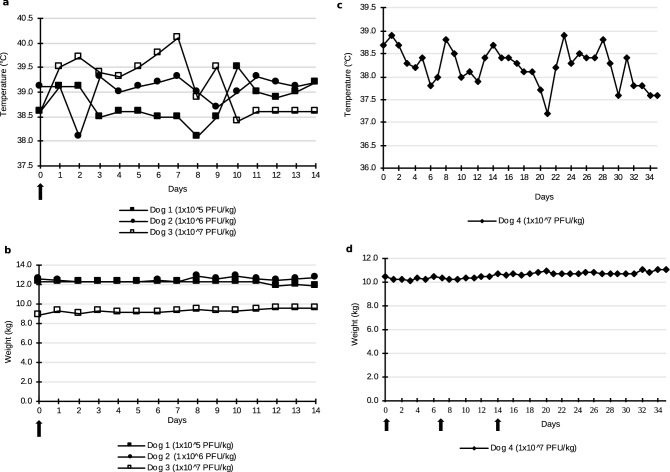
Temperature and weight of dogs after single or three intravenous injections of TG6002. (**a)** Temperature of dogs after a single intravenous injection of TG6002. (**b)** Weight of dogs after a single intravenous injection of TG6002. (**c)** Temperature after three intravenous injections of TG6002. (**d)** Weight after three intravenous injections of TG6002. No significant modification of weight and temperature was noticed after single or repeated intravenous injections of TG6002. Arrows indicate TG6002 administrations.

Three healthy dogs were injected intravenously with escalating doses of TG6002. During this single injection phase, hyperthermia scored as grade 1 at days 2 (39.7 °C) and 6 (39.8 °C) and as grade 2 at day 7 (40.1 °C) for the dog treated at 1 × 10^7^ PFU/kg were noticed (Fig. 2a). For the two other dogs which received 10^5^ and 10^6^ PFU/kg of TG6002, no hyperthermia was observed (Fig. 2a). No other clinical abnormalities, particularly, no decrease in body weight were recorded for any of the dogs (Fig. 2b).

As the MTD was not reached, the highest tested dose (1 × 10^7^ PFU/kg) was selected to be administered in the second phase of the study (Fig. 1).

During the repeated injection phase, no hyperthermia or decrease in body weight was noticed (Fig. 2c,d). From day 27 through day 34, vesicles on the mucosal side of the upper lips were observed. No other clinical abnormalities were noticed.

**Figure 3:**
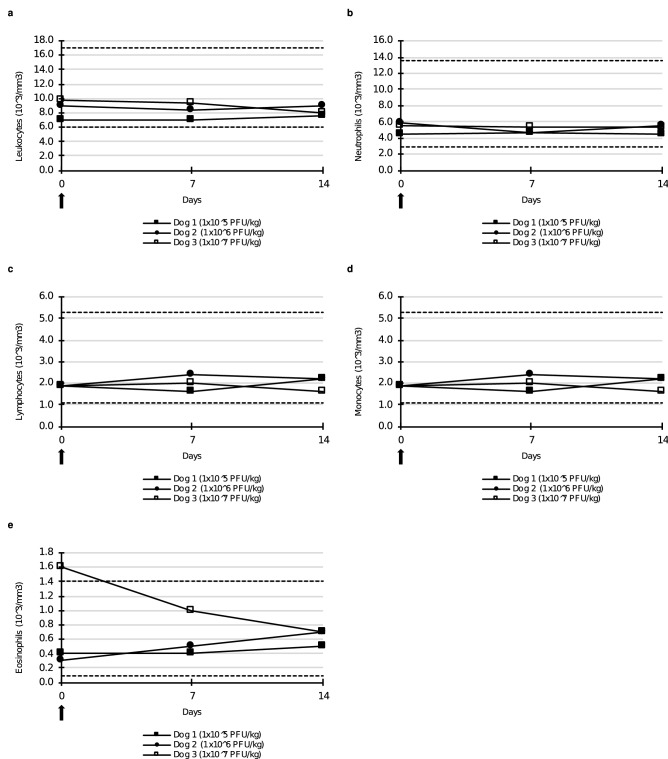
White blood cell count of dogs after a single intravenous injection of TG6002. (**a)** Leukocytes. (**b)** Neutrophils. (**c)** Lymphocytes. (**d)** Monocytes. (**e)** Eosinophils. (**f)** Basophils. TG6002 did not induce significant changes in any blood cell counts after a single injection. Arrows indicate TG6002 administrations. Dotted lines represent reference intervals.

**Figure 4:**
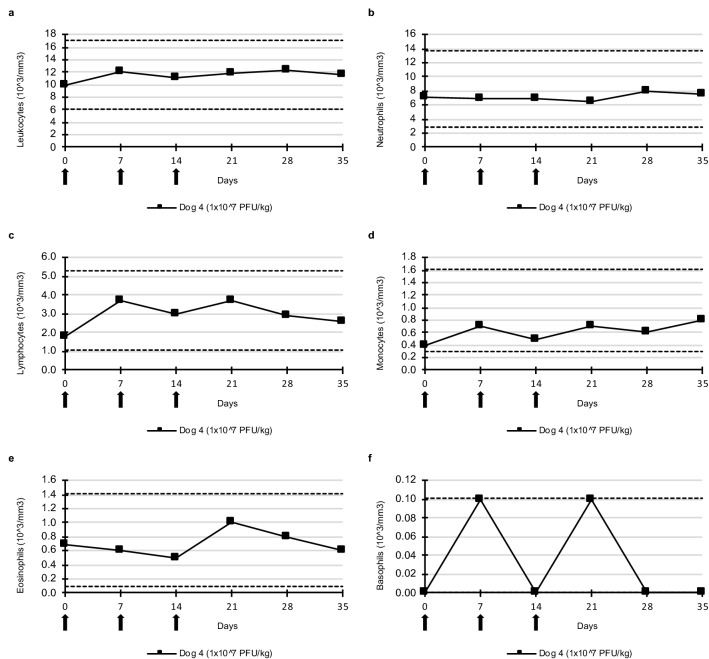
White blood cell count after three intravenous injections of TG6002. (**a)** Leukocytes. (**b)** Neutrophils. (**c)** Lymphocytes. (**d)** Monocytes. (**e)** Eosinophils. (**f)** Basophils. TG6002 did not induce significant changes in any blood cell counts after repeated injections at 1 × 10^7^ PFU/kg. Arrows indicate TG6002 administrations. Dotted lines represent reference intervals.

Hematology did not reveal any obvious changes for both parts of the study (Figs. 3, 4).

At inclusion in the single injection protocol, a slight increase of blood urea nitrogen (grade 1) for Dog 1 and a slight increase of glucose (grade 1) for Dog 2 and Dog 3 which normalized at day 7 and 14 were noticed (Table 1). For Dog 1, Dog 2 and Dog 3, total protein count was 5 to 15% below the reference range during the first phase of the study. For the dog receiving three injections of TG6002, only a slight decrease (< 5%) of total protein count was noticed at days 7, 14, 21, 28 and 35 (Table 1).

**Table 1:** Biochemical analyses of dogs after single and three intravenous injections of TG6002.

Dog	Day	Blood urea nitrogen (mmol/L)	Albumin (g/L)	Protein (g/L)	ALAT (UI/L)	ALP (UI/L)	Cholesterol (mmol/L)	Glucose (mmol/L)
Dog 1 (1 × 10^5^ PFU/kg)	D0	**7.1**	30	55	73	59	3.9	5.9
	D7	2.4	29	**49**	69	48	3.8	5.6
	D14	2.7	31	**47**	61	58	4.1	5.9
Dog 2 (1 × 10^6^ PFU/kg)	D0	3.2	28	**45**	32	37	3.0	**6.7**
	D7	3.1	31	**47**	49	56	3.8	5.9
	D14	4.0	31	56	52	60	3.6	5.4
Dog 3 (1 × 10^7^ PFU/kg)	D0	4.4	29	**45**	41	57	4.1	**6.1**
	D7	4.6	28	**51**	33	75	4.5	5.7
	D14	5.6	30	**53**	38	69	5.0	5.7
Dog 4 (3 injections 1 × 10^7^ PFU/kg)	D0	3.4	31	56	25	47	4.3	5.8
	D7	3.5	28	**52**	24	68	4.3	4.9
	D14	4.2	28	**54**	29	86	4.3	5.0
	D21	4.7	27	**53**	23	81	3.8	4.0
	D28	4.0	28	55	23	70	4.4	4.5
	D35	4.1	29	**52**	23	82	4.6	5.4
Reference value		2.0–7.0	25–40	55–75	5–80	5–200	3.0–7.0	3.0–6.0

Bold numbers refer to values that are outside the reference range.

### Biodistribution and virus shedding

#### Blood, urine, feces, and saliva

Viral DNA was detected in blood one hour after a single (Dog 3) or multiple (Dog 4) injections of TG6002 at 1 × 10^7^ PFU/kg (Table 2). For Dog 3 and Dog 4, the amount of virus detected in blood after one hour represented less than 1% of the virus injected. All other samples obtained at the lower doses of infection or other sampling times gave results below the limit of the assay detection.

**Table 2:** Detection of vaccinia virus DNA in blood by q-PCR assay after single and multiple intravenous injection of TG6002.

Dog	Time of sampling	Mean (± SD) (VG/ml)
Dog 1 (1 × 10^5^ PFU/kg)	Day 0	< LOD
	Day 0 + 1 h	< LOD
	Day 3	< LOD
	Day 7	< LOD
	Day 10	< LOD
	Day 14	< LOD
Dog 2 (1 × 10^6^ PFU/kg)	Day 0	< LOD
	Day 0 + 1 h	< LOD
	Day 3	< LOD
	Day 7	< LOD
	Day 10	< LOD
	Day 14	< LOD
Dog 3 (1 × 10^7^ PFU/kg)	Day 0	< LOD
	Day 0 + 1 h	60,200 (± 20,700)
	Day 3	< LOD
	Day 7	< LOD
	Day 10	< LOD
	Day 14	< LOD
Dog 4 (3 injections—1 × 10^7^ PFU/kg)	Day 0	< LOD
	Day 0 + 1 h	17,300 (± 7570)
	Day 3	< LOD
	Day 7	< LOD
	Day 7 + 1 h	29,500 (± 14,600)
	Day 10	< LOD
	Day 14	< LOD
	Day 14 + 1 h	32,200 (± 10,700)
	Day 17	< LOD
	Day 21	< LOD
	Day 24	< LOD
	Day 28	< LOD
	Day 31	< LOD
	Day 35	< LOD

All samples were measured in triplicate.

*LOD* limit of detection, *SD* standard deviation, *VG* viral genome.

Viral DNA was not detected in urine, feces and saliva in the first part of the study (days 0, 7 and 14) nor in the second part (days 0, 7, 14, 21, 28, 35) of the study.

For Dog 4 treated with three injections of TG6002 at 1 × 10^7^ PFU/kg, viral DNA was not detected in the vesicles noticed on the mucosal side of the upper lips between days 27 to 34.

### Organ samples

Fourteen days after a single intravenous injection of TG6002, viral DNA was not detected by q-PCR assay in samples from the heart, liver, mesenteric and pre-scapular lymph nodes, kidneys, spleen, lungs and testicles. In each case, the results were below the limit of the assay’s detection determined at 40 copies/10µL of the extracted sample (Table 3).

**Table 3:** Detection of vaccinia virus DNA in organs by q-PCR assay after single and multiple intravenous injection of TG6002.

Dog	Sample	Mean (± SD) (VG/mg)
Dog 1 (1 × 10^5^ PFU/kg)	Spleen	< LOD
	Liver	< LOD
	Kidney	< LOD
	Testicle	< LOD
	Mesenteric lymph node	< LOD
	Prescapular lymph node	< LOD
	Lung	< LOD
	Heart	< LOD
Dog 2 (1 × 10^6^ PFU/kg)	Spleen	< LOD
	Liver	< LOD
	Kidney	< LOD
	Testicle	< LOD
	Mesenteric lymph node	< LOD
	Prescapular lymph node	< LOD
	Lung	< LOD
	Heart	< LOD
Dog 3 (1 × 10^7^ PFU/kg)	Spleen	< LOD
	Liver	< LOD
	Kidney	< LOD
	Testicle	< LOD
	Mesenteric lymph node	< LOD
	Prescapular lymph node	< LOD
	Lung	< LOD
	Heart	< LOD
Dog 4 (3 injections—1 × 10^7^ PFU/kg)	Spleen	155 (± 269)
	Liver	< LOD
	Kidney	< LOD
	Testicle	< LOD
	Mesenteric lymph node	< LOD
	Prescapular lymph node	< LOD
	Lung	< LOD
	Heart	< LOD

All samples were measured in triplicate.

*LOD* limit of detection, *SD* standard deviation, *VG* viral genome.

Thirty-five days after the first injection, small amounts of viral DNA (1.55 × 10^2^ VG/ml; SD 2.60 × 10^2^ VG/ml) were detected in the spleen of Dog 4 treated with three injections. Splenic viral DNA quantification was near the detection limit (40 copies/10µL) of the assay (Table 3). Viral DNA was not detected in the heart, liver, mesenteric and pre-scapular lymph nodes, kidneys, lungs and testicles of Dog 4 by q-PCR assay.

### Immune responses

**Figure 5:**
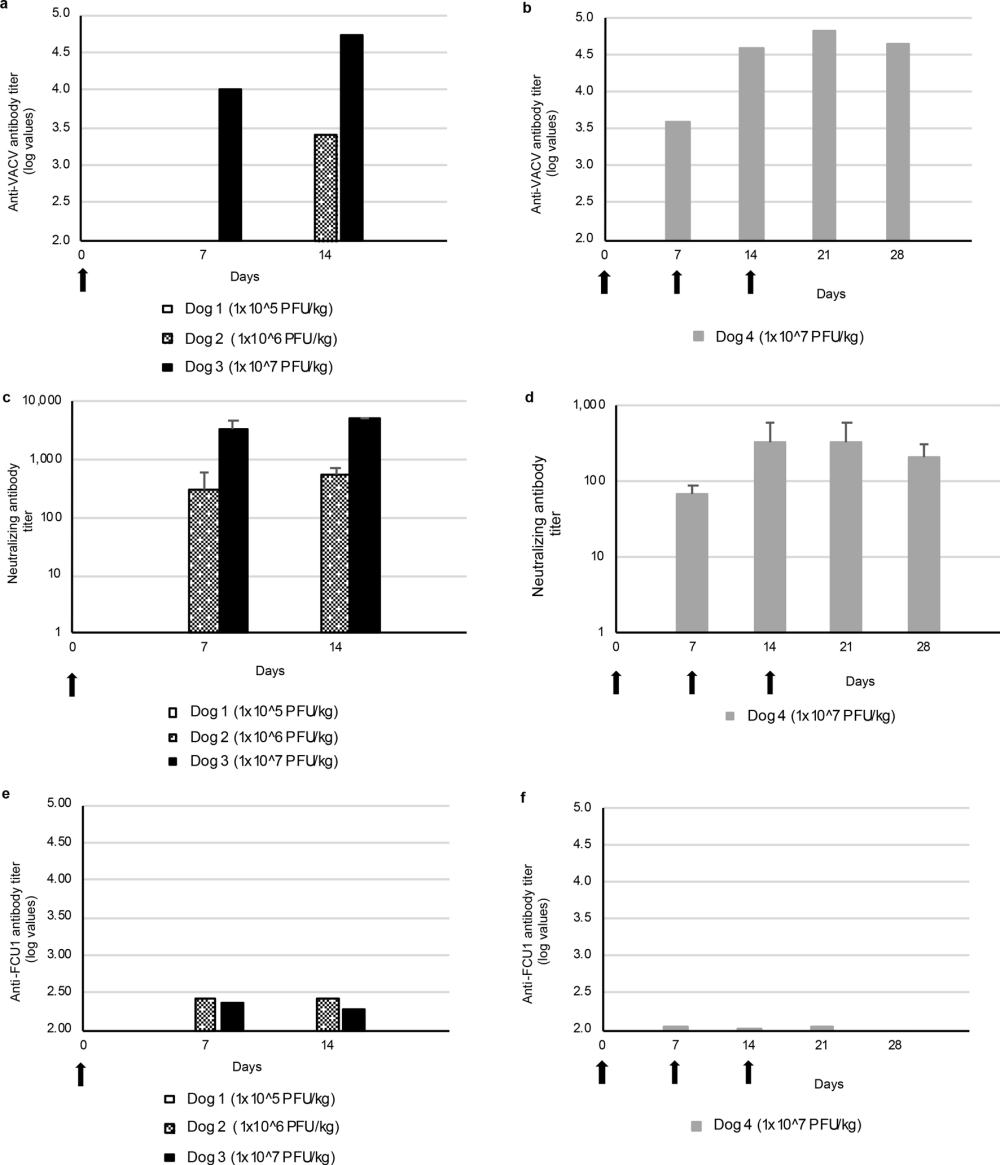
Immune responses of dogs after single or multiple intravenous injection of TG6002. (**a)** Anti-VACV antibody titers after single injection of TG6002. (**b)** Anti-VACV antibody titers after three injections of TG6002. (**c)** Neutralizing antibody titers after single injection of TG6002. (**d)** Neutralizing antibody titers after three injections of TG6002. (**e)** Anti-FCU1 antibody titers after single injection of TG6002. (**f)** Anti-FCU1 antibody titers after three injections of TG6002. Arrows indicate TG6002 administrations. For neutralizing antibody titers, samples were measured in triplicate and data are shown as mean ± SD.

Anti-VACV antibodies were not detected after one injection at 1 × 10^5^ PFU/kg at days 7 and 14 (Fig. 5a). However, 14 days after one injection at 1 × 10^6^ PFU/kg, a low level of anti-VACV antibodies was detected (Fig. 5a). Furthermore, after one injection at 1 × 10^7^ PFU/kg of TG6002, anti-VACV antibodies were detected seven days after injection with a slight increase in level at day 14 (Fig. 5a). After three injections at 1 × 10^7^ PFU/kg of TG6002, anti-VACV antibodies were detected from days 7 through 28 (Fig. 5b). The highest level of anti-VACV antibodies was reached seven days after the third injection.

Neutralizing antibodies against VACV were detectable by day 7 for dogs receiving single and multiple injections. Thus, the virus dose did not affect the time required for the development of neutralizing antibodies. For dogs receiving a single dose injection of TG6002, higher titers of neutralizing antibodies were noticed with higher dose of TG6002 (Fig. 5c). For the dog receiving three injections, an increase of neutralizing antibody titers was measured up until fourteen days with a decrease after that (Fig. 5d).

Anti-FCU1 antibodies were not detected after one injection at 1 × 10^5^ PFU/kg at days 7 and 14 (Fig. 5e). Low levels of anti-FCU1 antibodies were noticed for both dogs treated with a single injection of TG6002 at 1 × 10^6^ PFU/kg or 1 × 10^7^ PFU/kg. The levels of anti-FCU1 antibodies were not related to the dose injected. Similarly, a low level of anti-FCU1 antibodies was measured at days 7, 14 and 21 for the dog treated with three injections of TG6002 and these anti-FCU1 antibodies could not be detected at day 28 (Fig. 5f).

## Discussion

This study describes the clinical toxicity, viral shedding and immune response after intravenous administration of TG6002 in four healthy immune-competent dogs. The data collected indicate that the MTD was not reached even at the highest tested dose of 1 × 10^7^ PFU/kg. In human clinical trials, a dose of 10^9^ PFU of VACV (corresponding approximately to 10^7^ PFU/kg) is generally administered by intravenous route^9,50^. The administration of TG6002 was well tolerated for all dogs. Only a fluctuating grade 2 hyperthermia was noticed during one week for the dog receiving one injection of TG6002 at 1 × 10^7^ PFU/kg. No hyperthermia was observed for the dog with three injections at the same dosage. Hyperthermia is a well-known clinical sign associated with viral infection and frequently reported in oncolytic virotherapy^9,11,14,56^. Therefore, the fluctuating hyperthermia observed in Dog 3 might have been induced by VACV administration. More blood viral DNA was detected at one hour for Dog 3 compared to Dog 4, an observation supporting this hypothesis. A previous study also revealed transient hyperthermia for one dog after intramuscular administration of TG6002^57^. In human trials, adverse events secondary to intratumoral or intravenous administration of oncolytic VACV included hyperthermia, rigors, abdominal pain, nausea, vomiting, tiredness and headache^13,14,49,56,58^. VACV infection is generally associated with cutaneous pock lesions. For Dog 4 receiving 3 intravenous injections, transient oral pustules were observed 27 days after the first viral injection (13 days after the last injection). No viral DNA was isolated in pustules of Dog 4. As q-PCR analysis is more sensitive than plaque assay analysis, q-PCR was preferred to detect the presence of the virus in the pustules^59,60^. Moreover, due to the absence of viral genome and the negative impact of oral bacteria on cell culture, plaque assay analysis was not performed. Human patients diagnosed with cancer receiving attenuated oncolytic VACV have been reported to develop mucocutaneous pustules a few days after treatment^9,12,14,49–52^. In a few cases, PCR analysis confirmed that pustular lesions were induced by VACV^49,51^. After a few weeks, the lesions generally resolved without complications and patients did not develop systemic or recurrent disease. Moreover, for all cases reported, the presence of a few pustules did not require the interruption of the treatment. Due to ethical concerns, safety studies evaluating oncolytic viruses only involve small numbers of healthy animals. Considering the low number of dogs in our study, it is important to remain cautious about the absence of major toxicities.

Oncolytic virus administration can be associated with hematological changes. Indeed, a decrease in lymphocytes, platelets, red blood cells or hemoglobin and an increase in neutrophils was reported in liver cancer patients treated with a *TK* deleted oncolytic VACV encoding GM-CSF^11,14^. However, safety evaluation of *TK* and *vaccinia growth factor* (*VGF*) deleted oncolytic VACV encoding CD40 ligand intravenously administered in dogs did not reveal hematological changes^61^. In our study, hematological examinations and biochemistry analyses did not reveal changes for dogs receiving single or repeated administrations. Only a transient slight increase of basophil count was noticed for dogs treated at 10^7^ PFU/kg and was suspected to be a physiological variation or induced by TG6002. A previous study evaluating the safety of intramuscular injections of TG6002 did not reveal hematological or biochemical adverse events^57^. In our study, no major clinical or hematological adverse events were observed. TG6002 is expected to replicate within tumors. In these healthy dogs, the absence of tumors may lead to a lack of replication of VACV, thereby limiting the viral load in blood. In addition to this safety study, tolerance will have to be evaluated in a clinical trial with dogs diagnosed with cancer. Furthermore, rapid clearance of VACV by the intravenous route could also explain the absence of adverse events in healthy dogs.

To assess the pharmacokinetics, blood draws were performed after TG6002 administration. Pharmacokinetics of TG6002 were dose-related. Viral DNA was only detected in the dogs’ blood one hour after a single injection of TG6002 at 1 × 10^7^ PFU/kg and multiple injections of TG6002 at 1 × 10^7^ PFU/kg. As 50 viral genomes account for about 1 PFU, viral DNA detected at 1-h amounts to less than 1% of the injected dose. Therefore, healthy dogs appeared to clear the virus quickly, limiting its ability to induce adverse events. However, rapid clearance of OV can also impede the targeting of metastases. Earlier assessment, within one hour, associated with assessment of infectivity would have been interesting. In dogs, evaluation of viral DNA after intravenous injection of a *TK* and *VGF* deleted oncolytic VACV encoding CD40 ligand revealed that viral load decreased during the first four hours following administration but was still detectable at a low titer, one week after injection^61^. For those dogs, infectious virus in blood was only detectable directly after virus administration^61^. In human clinical trials using intravenous oncolytic VACV, a dose-related pharmacokinetic profile with rapid clearance of the virus was also reported^12,49^. The majority of viral genomes were cleared by four hours after single intravenous administration of a Western Reserve strain oncolytic VACV at 3 × 10^8^ PFU to 3 × 10^9^ PFU^49^. A significant decrease in viral genome was also observed 4 h after intravenous injection of Pexa-Vec at dose levels of 1 × 10^6^, 1 × 10^7^, or 3 × 10^7^ PFU/kg in patients with colorectal cancer^12^. Moreover, Park et *al.* did not detect the presence of infectious VACV in blood two hours after intravenous administration of Pexa-Vec at 3 × 10^7^ PFU/kg^12^. The innate immune system could participate in the rapid clearance of VACV. Complement participates in the innate immune system, acting to target foreign pathogens for opsonization, neutralization, phagocytosis, and clearance from the circulatory system^62^. Evgin et al*.*, showed that inhibition of complements C1 and C3 prevented viral neutralization in both naive and immune plasma samples^63^. As TG6002 is expected to replicate within tumors, it is not surprising that a delayed viremia was not observed in healthy dogs. A secondary viremia peak, supposed to be tumor viral release, has been observed in several clinical trials involving patients receiving oncolytic VACV^11,14,58^.

Viral DNA was only detected in the spleen of the dog with repeated injections. As q-PCR analysis is more sensitive than immunohistochemistry analysis, q-PCR was preferred. Plaque assay analyses would have been interesting to assess infectivity of the virus. For other organs, in particular those with dividing cells like testicles, no viral DNA was detected. A previous study evaluating intravenous injections of TG6002 at 1 × 10^7^ PFU on subcutaneous human colon cancer in nude mice reported higher VACV titers in tumors and very low amounts of VACV in some organs^26^. Similar findings have been observed in healthy dogs treated with intravenous double-deleted oncolytic VACV encoding CD40 ligand^61^. As previously described, viruses can be opsonized by antibodies or other serum proteins, leading to the degradation of the vector through the reticuloendothelial system of red pulp macrophages in the spleen and Kupffer cells of the liver^44,61,64^. Sequestration of the viral vector by the mononuclear phagocytic system in the liver and spleen can lead to a reduced oncolytic activity and explain the absence of detectable VACV at autopsy on days 14 and 35^44^.

Biosafety is a major issue with OV. In particular, VACV is known to remain infectious for a long time in urine, feces or the environment^53–55^. Safety studies on beagle dogs receiving *TK* and *VGF* deleted oncolytic VACV encoding CD40 ligand reported small amounts of viral DNA, but no infectious virus, in urine and saliva after intravenous injections^61^. In patients with cancer receiving oncolytic VACV, detection of infectious virus is rare^11,65^. Only viral DNA has been identified and suspected to be DNA fragments from virus digested by leukocytes in urine, saliva and feces of patients^52,56^. In the present study, no viral genome copies were detectable in urine, saliva and feces. Since the dogs in this study did not have cancer, the viral load could probably be higher in dogs with tumors as they allow viral amplification. Thus, tolerance and viral shedding will have to be evaluated in neoplastic dogs.

TG6002 injections induce immune responses, including VACV antibodies, antibodies against transgene and OV-neutralizing antibodies. VACV antibodies were expected after TG6002 injections, even without VACV replication. A low level of anti-FCU1 antibodies was noticed in our study. This could be explained by low immunogenicity of the FCU1 protein or by the absence of replication of VACV in healthy dogs leading to poor expression of the FCU1 protein. Considering VACV, neutralizing antibodies can be observed at baseline secondary to vaccination or induced by the OV therapy. The development of neutralizing antibodies within 7 days after viral exposure is reassuring from a biosafety perspective. Indeed, neutralizing antibodies could prevent the development of systemic adverse events induced by the viral vector. However, the use of a virus with no relationship to the host, such as VACV for dogs, may represent a limit of this model considering the absence of pre-existing immunity. Indeed, the development of antiviral neutralizing antibodies and VACV antibodies are expected to limit the systemic OV delivery, rendering repeat systemic treatments ineffective. However, in several oncolytic VACV clinical studies, antitumor responses have been described despite the presence of anti-vaccinia antibodies at baseline, due to previous VACV vaccination in the World Health Organization’s smallpox eradication campaign, and despite the increase of neutralizing antibodies during the study^9,11,14,66^. VACV produces a unique form of virus particles called extracellular enveloped virus, which can evade the neutralizing antibodies and make this virus suitable for systemic and repeated administrations^67^.

This study provides strong evidence that intravenous injections of TG6002 is well tolerated in healthy dogs. Furthermore, the evaluation of viral shedding did not reveal viral excretion in the environment. These results, combined with previous data, support the evaluation of systemic administration of TG6002 in both humans and dogs with diagnosed cancers. This approach fits in a “One Health—One Medicine” concept and may contribute to the development of new therapies for animal and human cancers.

## Methods

### Viral vectors and cells

Oncolytic VACVs were derived from the Copenhagen strain and are deleted of both the thymidine kinase (*J2R*) gene and the large subunit of ribonucleotide reductase (*I4L*) gene. TG6002 expressing the fusion gene *FCU1* (Δ*I4L*Δ*J2R*/FCU1 VACV) was constructed as previously described^26^. The same methods were used to generate the double-deleted VACV expressing GFP, designated VVTG17990, by homologous recombination between the previously described Δ*J2R*/GFP VACV and the pΔI4L shuttle plasmid containing the selection cassette encoding the guanine phosphoribosyltransferase surrounded by the flanking sequences of VACV *I4L* gene^68^. TG6002 and VVTG17990 were amplified in chicken embryonic fibroblasts (CEF).

Primary CEF were used for recombination, amplification, and production of viral vectors. CEF cells were prepared and maintained as previously described^69^.

### Laboratory dogs

Four healthy adult male beagle dogs (Harlan Laboratories, Gannat, France) with no oncolytic virus pre-exposure were used. All dogs were acclimatized for seven days and were under the care of a veterinarian. The dogs were housed individually in inox-steel bar boxes with a resin soil substrate and a softwood chips litter. The room temperature was 19 °C (+ /− 2 °C) with a humidity greater than 35%, and the day/night cycle was 12:12 h. Dogs were fed daily with a commercial diet and given potable water ad libitum.

### Study design and treatments

The first part of the study aimed to determine the maximum tolerated dose (MTD) in three dogs, with each dog receiving an increasing dose of TG6002 by intravenous injection (Fig. 1). The second part of the study assessed tolerability of several intravenous injections of TG6002 at the identified MTD in one dog (Fig. 1). In the first part of the study, three dogs were treated on day 0 with a single intravenous injection of TG6002. TG6002 was diluted in 100 mL of NaCl 0.9% and perfused, under sedation, in the cephalic vein for one hour. Dogs were sedated by intravenous administration of 0.2 mg/kg butorphanol (Torbugesic, Zoetis, Malakoff, France) and 10 µg/kg medetomidine (Domitor, Orion Corporation, Espoo, Finland). The doses chosen in this study were similar to the doses previously described in the safety study by intramuscular route^34^. Dog 1 received 1 × 10^5^ PFU/kg, Dog 2 received 1 × 10^6^ PFU/kg, Dog 3 received 1 × 10^7^ PFU/kg. To detect any side effects, injections were performed early in the morning to allow for observations and administration between each dog were spaced by 7 days. Dogs were evaluated daily by a physical examination for 14 days after the injection of TG6002. Complete blood counts and biochemistry analyses were performed before TG6002 injection (day 0) and at 7 and 14 days afterward.

To measure viral shedding, blood samples were collected before injection (day 0), one hour after injection, and at days 3, 7, 10 and 14; saliva, urine and feces were collected at days 0 (before injection), 7 and 14.

The MTD was defined as the highest dose of TG6002 that did not cause major side effects.

One dog (Dog 4) was used for evaluating the tolerability of multiple injections. Dog 4 received three intravenous injections of TG6002 at days 0, 7 and 14 at the defined MTD. TG6002 was administered according to the same procedure. Dog 4 was evaluated daily by a physical examination for 35 days. Complete blood counts and biochemistry analyses were performed at days 0, 7, 14, 21, 28 and 35.

Blood samples were collected at days 0 (before first injection), 3, 7 (before second injection), 14 (before third injection), 17, 21, 24, 28, 31, 35 and one hour after each intravenous virus administration (days 0, 7, 14). Saliva, urine and feces were collected at days 0 (before first injection), 7 (before second injection), 14 (before third injection), 21, 28 and 35.

At the end of the study (day 14 for Dog 1, Dog 2 and Dog 3; day 35 for Dog 4), dogs were euthanized to assess virus biodistribution. The dogs were anesthetized with an intravenous administration of 0.2 mg/kg of butorphanol (Torbugesic, Zoetis, Malakoff, France), 3 mg/kg of ketamine (Ketamine 1000, Virbac, Carros, France) and 15 µg/kg of medetomidine (Domitor, Orion Corporation, Espoo, Finland). After each dog was sedated, an intravenous injection of 180 mg/kg of sodium pentobarbital solution (Dolethal, Vetoquinol, Magny Vernois, France) was given. Death was confirmed by the inability to hear respiratory sounds and heartbeat using a stethoscope. Samples of heart, liver, mesenteric and pre-scapular lymph nodes, kidneys, spleen, lungs, and testicles were collected from all dogs and stored at − 80 °C until analysis.

### Adverse events

Adverse events were monitored by daily physical examination, complete blood count and biochemistry analyses and graded according to the Veterinary Cooperative Oncology Group Common Terminology Criteria for Adverse Events guidelines^70^.

### Complete blood count and biochemistry analysis

Complete blood counts were performed using a Procyte Hematology analyzer (IDEXX Laboratory Inc, Westbrook, ME). Biochemistry analyses were performed using a Catalyst Biochemistry analyzer (IDEXX Laboratory Inc, Westbrook, Maine, United States).

### Sample collection for viral shedding

Five milliliters of blood was collected in an EDTA tube, saliva samples were taken with buccal swabbing (Universal viral transport kit, Becton Dickinson, Franklin Lakes, New Jersey, United States), 5 ml of urine was collected in a sterile Falcon tube, and one gram of feces was transferred to a sterile Falcon tube. Samples were stored at − 80 °C until analysis.

### q-PCR

Quantitative polymerase chain reaction was used to detect TG6002 genomes in whole blood, saliva, urine, feces and organ samples. DNA was extracted from 100 µl of whole blood, saliva and urine. For organs and feces, 30 mg of each organ sample and one gram of feces were transferred in GentleMACS M–type tubes (Miltenyi Biotec, Bergisch Gladbach, Germany) containing 600 µL of PBS and were dissociated using a GentleMACS dissociator (Miltenyi Biotec, Bergisch Gladbach, Germany). DNA was extracted from one hundred microliters of lysates. q-PCR was performed as previously described^34^. Samples were measured in triplicate. The limit of detection for analysis was 15 copies/100 µl for whole blood samples, 30 copies/100 µl for urine, 3600 copies/g for feces and 400 copies/30 mg for organ samples. The limit of detection for saliva could not be set due to the small amount collected.

### Enzyme-linked immunosorbent assay (ELISA)

For determination of anti-VACV antibody response, TG6002 was inactivated 20 min under UV lamp on ice. Ninety-six well plates (Corning, Corning, New York, United States) were coated with carbonate-bicarbonate buffer (Sigma, Saint Quentin, Fallavier, France) containing inactivated TG6002 (3 × 10^6^ PFU/100 µl/well) and allowed to attach overnight at 4 °C. Wells were washed with PBS and binding sites were blocked at room temperature for one hour with PBS containing 0.05% Tween 20 and 5% non-fat dry milk, followed by adding a twofold dilution series of the serum. After a two hours incubation at room temperature, plates were washed with PBS and 100 µl of recombinant peroxidase-conjugated, protein A/G (A/G-HRP protein, Thermo Fisher Scientific, Waltham, Maine, United States) diluted 20,000-fold was added. Plates were incubated for one hour at room temperature and washed with PBS. A volume of 100 µL of TMB solution (Sigma, Saint Quentin, Fallavier, France) was added for 30 min. The color reaction was stopped by 100 µL of 1 M H_2_SO_4_ solution. The absorbance was read at 450 nm on a spectrophotometric plate reader (Infinite M200 Pro, Tecan, Männedorf, Switzerland). A 1/3,000 diluted rabbit polyclonal VACV antibody (B65101R, Interchim Inc, Montlucon, France) was used as positive control. End-point-titers were determined as the highest dilution with an absorbance value greater than the absorbance value from normal dog sera. Results were given as log values and a log titer value of 2 or below was considered negative.

To determine the anti-FCU1 antibody response, ELISA was performed as described above except that plates were coated overnight with carbonate-bicarbonate buffer containing FCU1 peptide at 7.6 ng/well. A 1/3000 diluted rabbit polyclonal anti-FCU1 peptide was used as positive control^69^.

### Neutralizing antibodies

Two-fold serial dilutions from 1/40 to 1/5120 of serum samples were incubated with 2 × 10^3^ PFU of VVTG17990 in Corning tubes for one hour at 37 °C and incubated with CEF for 3 days at 37 °C with 5% CO_2_. Plates were examined using fluorescence microscopy (Stereoscopic microscope Nikon SMZ18 and epi-fluorescence light source Nikon Intensilight C-HGFI) to score GFP positive plaques. The percent neutralization was calculated relative to the number of GFP plaques in the absence of serum. The neutralizing antibody titer was defined as the highest serum dilution resulting in a 50% reduction in the number of plaques. Samples were measured in triplicate and mean neutralization titers for dogs were plotted ± standard deviation.

### Ethical approval

This study was conducted in accordance with European legislation and French regulations on the protection of animals used for scientific purposes (Directive 2010/63/EU, 2010; Code rural, 2018; Décret 2013–118, 2013) and complied with the recommendations of the “Charte nationale portant sur l’éthique en expérimentation animale” established by the “Comité National de Réflexion Ethique sur l’Expérimentation Animale” (Ministère de l'Enseignement Supérieur, de la Recherche et de l'Innovation—Ministère de l’Agriculture et de l’Alimentation). The protocol (No. 1431_v2) was approved by the VetAgro Sup Ethical Committee (C2EA No. 18) and the Ministry of National Education, Higher Education and Research. The study was carried out in compliance with the ARRIVE guidelines.

## Data Availability

The authors state that there is not any restriction on the availability of materials or information.
